# Supplementary material for: Acute stress promotes effort mobilization for safety-related goals
Source: Commun Psychol. 2024 Jun 1;2:50. doi: 10.1038/s44271-024-00103-7 (PMC11332123; doi:10.1038/s44271-024-00103-7)
Supplement: Supplementary file 2 — Supplementary Materials [file 44271_2024_103_MOESM2_ESM.pdf]

Supplementary Materials

Supplementary Figures

Supplementary Figure 1: No evidence for a stress-induced changes in rate of change in force applied

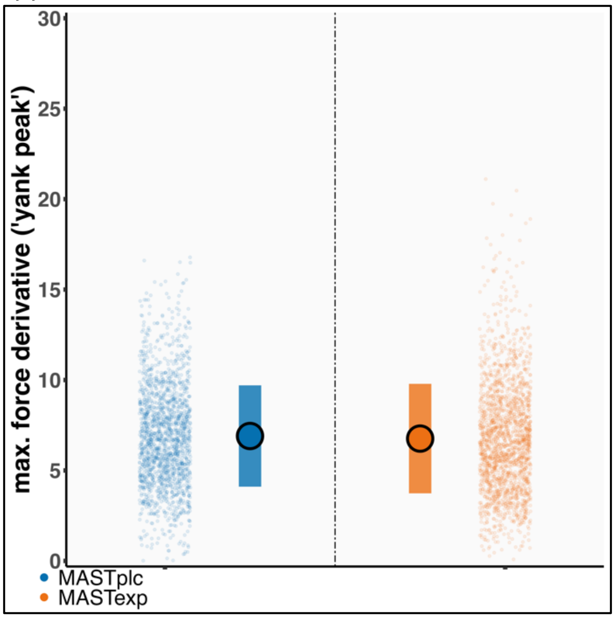

Supplementary Fig. 1 Legend. MAST$_{EXP}$ and MAST$_{PLC}$ participants did not significantly differ in the rate of change in force applied (yank peak, higher y-axis value = greater rate of change in force) following the decision to exert effort to avoid the threat-of-shock. Trial-level raw data (individual data points) and group-level summaries of individual-level averaged raw data (mean+error bars) are both shown. Error bars represent SD. n=80 participants.

Supplementary Tables

Supplementary Table 1: Experiment 1 Participant Characteristics

Variable	$MAST_{PLC}$	$MAST_{EXP}$	Test Statistic	p-value	Effect size	Effect size 95% CI
**Sex**	27 F/13 M	30 F/10 M	0.24[a]	0.62	0.06[a]	0.00 to 0.27
**Oral contraceptive**	5 Y/22 N	5 Y/25 N	0.00[a]	>0.999	0.06[a]	0.00 to <0.01
**Luteal Phase[c]**	12 Y/7 N	9 Y/12 N	0.93[a]	0.33	0.15[a]	0.00 to 0.46
**Age**	21.10 (2.64)	21.93 (2.70)	-1.38[b]	0.17	-0.31[b]	-0.78 to 0.11
**BMI**	21.63 (2.06)	22.26 (2.55)	-1.21[b]	0.23	-0.27[b]	-0.70 to 0.17
**PSS (sum)**	31.42 (3.64)	31.20 (3.16)	0.29[b]	0.77	0.07[b]	-0.38 to 0.54
**BIS (mean)**	2.07 (0.26)	2.14 (0.24)	-1.20[b]	0.24	-0.27[b]	-0.71 to 0.21
**BAS Drive (mean)**	2.29 (0.58)	2.27 (0.44)	0.22[b]	0.83	0.05[b]	-0.41 to 0.49
**BAS Fun Seeking (mean)**	1.98 (0.54)	2.12 (0.59)	-1.14[b]	0.26	-0.26[b]	-0.73 to 0.17
**BAS Reward Resp. (mean)**	1.60 (0.38)	1.59 (0.39)	0.12[b]	0.91	0.03[b]	-0.42 to 0.49
**FPQ Minor Pain**	20.73 (5.99)	21.20 (6.10)	-0.35[b]	0.73	-0.08[b]	-0.51 to 0.35
**FPQ Severe Pain**	38.73 (5.15)	39.58 (4.65)	-0.78[b]	0.44	-0.17[b]	-0.60 to 0.28
**FPQ Medical Pain**	28.15 (7.60)	29.27 (7.38)	-0.67[b]	0.50	-0.15[b]	-0.61 to 0.28
**PCS Magnification**	5.03 (2.69)	5.38 (2.54)	-0.60[b]	0.55	-0.13[b]	-0.57 to 0.35
**PCS Rumination**	8.45 (3.50)	8.40 (3.63)	0.06[b]	0.95	0.01[b]	-0.42 to 0.46
**PCS Helplessness**	8.75 (3.50)	8.97 (4.49)	-0.25[b]	0.80	-0.06[b]	-0.46 to 0.44
**MEI Mental Energy**	31.73 (8.02)	29.42 (7.81)	1.30[b]	0.20	0.29[b]	-0.15 to 0.76
**MEI Social Motivation**	35.42 (3.82)	33.60 (4.29)	2.01[b]	0.05	0.45[b]	0.00 to 0.88
**MEI Physical Energy**	26.05 (3.86)	24.55 (3.19)	1.89[b]	0.06	0.42[b]	-0.03 to 0.86
**MGF**	1.37 (0.23)	1.30 (0.18)	1.50[b]	0.14	0.33[b]	-0.10 to 0.76
**Shock calibration**	5.21 (2.52)	4.40 (1.99)	1.60[b]	0.11	0.36[b]	-0.11 to 0.82

Supplementary Table 1. *[a]=χ^2/Cramer's *V*, [b]=t-statistic/Cohen's *d*, [c]=all participants with a regular menstrual cycle were asked if their next period was expected within the next 10 days. BMI = Body Mass Index, PSS = Perceived Stress Scale (10-item version)[1], BIS = Behavioural Inhibition Scale[2], BAS = Behavioural Activation Scale[2], FPQ = Fear of Pain Questionnaire (version 3/30 items)[3], PCS = Pain Catastrophising Scale (13-item version)[4], MEI = Motivation and Energy Inventory (26-item version)[5], MGF = maximal grip force.

Supplementary Table 2: Experiment 2 Participant Characteristics

Variable	$MAST_{PLC}$	$MAST_{EXP}$	Test Statistic	p-value	Effect size	Effect size 95% CI
**Sex**	32 F/10 M	31 F/11 M	0.00[a]	>0.999	<0.01[a]	0.00 to <0.01
**Oral contraceptive**	9 Y/23 N	11 Y/20 N	0.13[a]	0.72	0.04[a]	0.00 to 0.29
**Luteal Phase[c]**	10 Y/12 N	12 Y/13 N	0.00[a]	>0.999	<0.01[a]	0.00 to <0.01
**Age**	23.74 (3.09)	22.14 (3.19)	2.33[b]	0.02	0.51[b]	0.07 to 1.05
**BMI**	22.07 (2.33)	21.20 (2.41)	1.67[b]	0.10	0.37[b]	-0.08 to 0.83
**SHAPS (sum)**	34.48 (4.71)	34.60 (2.68)	-0.14[b]	0.89	-0.03[b]	-0.44 to 0.44
**PSS (sum)**	31.95 (3.41)	30.67 (2.71)	1.91[b]	0.06	0.42[b]	-0.02 to 0.87
**BIS (mean)**	2.10 (0.34)	2.09 (0.26)	0.12[b]	0.91	0.03[b]	-0.44 to 0.44
**BAS Drive (Mean)**	2.26 (0.46)	2.33 (0.50)	-0.62[b]	0.54	-0.14[b]	-0.60 to .31
**BAS Fun Seeking (mean)**	1.94 (0.49)	2.01 (0.44)	-0.64[b]	0.52	-0.14[b]	-0.56 to 0.28
**BAS Reward Resp. (mean)**	1.54 (0.36)	1.47 (0.31)	0.98[b]	0.33	0.21[b]	-0.22 to 0.64
**MGF**	1.25 (0.21)	1.25 (0.16)	-0.14	0.89	-0.03[b]	-0.51 to 0.37

Supplementary Table 2. *[a]=χ^2/Cramer's *V*, [b]=t-statistic/Cohen's *d*, [c]=all people with a regular menstrual cycle were asked if their next period was expected within the next 10 days. BMI = Body Mass Index, SHAPS = Snaith-Hamilton Pleasure Scale[6], PSS = Perceived Stress Scale[1], BIS = Behavioural Inhibition Scale[2], BAS = Behavioural Activation Scale[2], MGF = maximal grip force.

Supplementary Table 3: Complete output for key main text regression models

Experiment 1

Main effect of threat-of-shock, effort cost, and time on choice (accept effort ~ effort_lm + threat_lm + block + (1 + effort_lm + threat_lm + block | participant number))

	B	Std. Error	z-value	p-value
**(Intercept)**	1.68	0.25	7.00	<0.001
**effort_lm**	-1.47	0.12	-11.82	<0.001
**threat_lm**	2.21	0.14	16.32	<0.001
**block**	-0.09	0.06	-1.62	0.11

Interaction between threat-of-shock, effort cost, and condition on choice (accept effort ~ condition*effort_lm*threat_lm + (1 + effort_lm + threat_lm + condition | participant number))

	B	Std. Error	z-value	p-value
**(Intercept)**	1.02	0.32	3.17	0.002
**condition**	0.93	0.50	1.86	0.06
**effort_lm**	-1.43	0.17	-8.52	<0.001
**threat_lm**	2.09	0.17	12.32	<0.001
**condition:effort_lm**	-0.12	0.24	-0.49	0.62
**condition:threat_lm**	0.17	0.24	0.72	0.47
**effort_lm:threat_lm**	-0.10	0.06	-1.66	0.10
**condition:effort_lm:threat_lm**	-0.21	0.10	-2.18	0.029

Experiment 2

Main effect of reward, effort cost, and time on choice (accept effort ~ effort_lm + reward_lm + block + (1 + effort_lm + reward_lm + block | participant number))

	B	Std. Error	z-value	p-value
**(Intercept)**	5.91	0.47	12.50	<0.001
**effort_lm**	-2.18	0.18	-12.26	<0.001
**reward_lm**	3.66	0.26	13.56	<0.001
**block**	-0.05	0.08	-0.68	0.50

Interaction between reward, effort cost, and condition on choice (accept effort ~ condition*effort_lm*reward_lm + (1 + effort_lm + reward_lm + condition | participant number))

	B	Std. Error	z-value	p-value
**(Intercept)**	6.02	0.60	9.99	<0.001
**condition**	-0.39	0.91	-0.43	0.67
**effort_lm**	-2.45	0.31	-7.88	<0.001
**reward_lm**	3.68	0.38	9.65	<0.001
**condition:effort_lm**	-0.01	0.41	-0.03	0.98
**condition:reward_lm**	0.12	0.53	0.22	0.83
**effort_lm:reward_lm**	-0.43	0.16	-2.71	0.01
**condition:effort_lm:reward_lm**	0.07	0.20	0.33	0.74

Experiment 1 versus Experiment 2

Interaction between experiment and condition on choice (accept effort ~ experiment*condition + effort_lm + (1 + effort_lm + condition + study | pp_num))

	B	Std. Error	z-value	p-value
**(Intercept)**	2.02	0.24	8.52	<0.001
**experiment**	-0.93	0.29	-3.25	0.001
**condition**	0.45	0.35	1.30	0.19
**effort_lm**	-0.94	0.06	-15.65	<0.001
**experiment:condition**	-1.12	0.43	-2.62	0.009

Supplementary References

1. Cohen, S. Perceived stress in a probability sample of the United States. in *The social psychology of health* 31–67 (Sage Publications, Inc, Thousand Oaks, CA, US, 1988).
2. Carver, C. S. & White, T. L. Behavioral inhibition, behavioral activation, and affective responses to impending reward and punishment: The BIS/BAS Scales. *J. Pers. Soc. Psychol.* **67**, 319–333 (1994). https://doi.org/10.1037/0022-3514.67.2.319
3. McNeil, D. W. & Rainwater, A. J. Development of the Fear of Pain Questionnaire-III. *J. Behav. Med.* **21**, 389–410 (1998). https://doi.org/10.1023/a:1018782831217
4. Sullivan, M. J. L., Bishop, S. R. & Pivik, J. The Pain Catastrophizing Scale: Development and validation. *Psychol. Assess.* **7**, 524–532 (1995). https://doi.org/10.1037/1040-3590.7.4.524
5. Fehnel, S. E., Bann, C. M., Hogue, S. L., Kwong, W. J. & Mahajan, S. S. The development and psychometric evaluation of the motivation and energy inventory (MEI). *Qual. Life Res.* **13**, 1321–1336 (2004). https://doi.org/10.1023/b:qure.0000037502.64077.4d
6. Snaith, R. P. *et al.* A Scale for the Assessment of Hedonic Tone the Snaith–Hamilton Pleasure Scale. *Br. J. Psychiatry* **167**, 99–103 (1995). https://doi.org/10.1192/bjp.167.1.99
