# Supplementary material for: Acute stress promotes effort mobilization for safety-related goals
Source: Commun Psychol. 2024 Jun 1;2:50. doi: 10.1038/s44271-024-00103-7 (PMC11332123; doi:10.1038/s44271-024-00103-7)
Supplement: Supplementary file 3 — Reporting Summary [file 44271_2024_103_MOESM3_ESM.pdf]

nature portfolio

Corresponding author(s): Dennis Hernaus

Last updated by author(s): May 3, 2024

Reporting Summary

Nature Portfolio wishes to improve the reproducibility of the work that we publish. This form provides structure for consistency and transparency in reporting. For further information on Nature Portfolio policies, see our Editorial Policies and the Editorial Policy Checklist.

Statistics

For all statistical analyses, confirm that the following items are present in the figure legend, table legend, main text, or Methods section.

n/a	Confirmed	
☐	☒	The exact sample size (*n*) for each experimental group/condition, given as a discrete number and unit of measurement
☐	☒	A statement on whether measurements were taken from distinct samples or whether the same sample was measured repeatedly
☐	☒	The statistical test(s) used AND whether they are one- or two-sided<br>*Only common tests should be described solely by name; describe more complex techniques in the Methods section.*
☐	☒	A description of all covariates tested
☐	☒	A description of any assumptions or corrections, such as tests of normality and adjustment for multiple comparisons
☐	☒	A full description of the statistical parameters including central tendency (e.g. means) or other basic estimates (e.g. regression coefficient) AND variation (e.g. standard deviation) or associated estimates of uncertainty (e.g. confidence intervals)
☐	☒	For null hypothesis testing, the test statistic (e.g. *F*, *t*, *r*) with confidence intervals, effect sizes, degrees of freedom and *P* value noted<br>*Give P values as exact values whenever suitable.*
☐	☒	For Bayesian analysis, information on the choice of priors and Markov chain Monte Carlo settings
☐	☒	For hierarchical and complex designs, identification of the appropriate level for tests and full reporting of outcomes
☐	☒	Estimates of effect sizes (e.g. Cohen's *d*, Pearson's *r*), indicating how they were calculated

Our web collection on statistics for biologists contains articles on many of the points above.

Software and code

Policy information about availability of computer code

Data collection	PsychoPy 2012.2.0
Data analysis	R v4.3.1<br>Matlab v.2021b<br>SPM12 (spm_bms function)<br>All code can be found here: https://osf.io/7pukd/

For manuscripts utilizing custom algorithms or software that are central to the research but not yet described in published literature, software must be made available to editors and reviewers. We strongly encourage code deposition in a community repository (e.g. GitHub). See the Nature Portfolio guidelines for submitting code & software for further information.

Data

Policy information about availability of data

All manuscripts must include a data availability statement. This statement should provide the following information, where applicable:

- Accession codes, unique identifiers, or web links for publicly available datasets
- A description of any restrictions on data availability
- For clinical datasets or third party data, please ensure that the statement adheres to our policy

All data and analyses presented in this manuscript are accessible via the following link: https://osf.io/7pukd/.

Human research participants

Policy information about studies involving human research participants and Sex and Gender in Research.

Reporting on sex and gender	We collected data on (biological) sex specifically. These are self-reported data and reported in the paper. Other than checking whether the study arms (acute stress, control) did not significantly differ in terms of sex distribution, we did not use these data in any other analyses.
Population characteristics	Experiment participants were sampled from a university student population. Acute stress and control participants did not differ on calibrated maximum grip force, calibrated shock intensity tolerance, age, sex, anti-conception use, menstrual phase, and self-rated measures of motivation, chronic stress, avoidance, hedonic capacity and fear of pain. No participants had a (DSM-V) diagnosis of a mental health, neurological, cardiovascular, metabolic, or endocrine disorder. No data on race or ethnicity were collected.
Recruitment	Participants were recruited via flyers/pamphlets at all university campus locations and via posts on a (student-wide) SONA platform.
Ethics oversight	Maastricht University Ethics Review Committee Psychology and Neuroscience

Note that full information on the approval of the study protocol must also be provided in the manuscript.

Field-specific reporting

Please select the one below that is the best fit for your research. If you are not sure, read the appropriate sections before making your selection.

☐ Life sciences ☒ Behavioural & social sciences ☐ Ecological, evolutionary & environmental sciences

For a reference copy of the document with all sections, see nature.com/documents/nr-reporting-summary-flat.pdf

Behavioural & social sciences study design

All studies must disclose on these points even when the disclosure is negative.

Study description	A quantitative cross-sectional randomised between-subjects design (arm: acute stress or control condition).
Research sample	Maastricht University student population. We specifically aimed to collect data from healthy human participants without a (DSM-V) diagnosis of a mental health condition, neurological, cardiovascular, metabolic, or endocrine disorder. Experiment 1 sample: n=80, no-stress control (n=40, 27F/13M, age M=21.10, SD=2.64), acute stress (n=40, 30F/10M, age M=21.93, SD=2.70). Experiment 2 sample: n=84, no-stress control (n=42, 32F/10M, age M=23.74, SD=3.09), acute stress (n=42, 31F/11M, age M=22.14, SD=3.19).
Sampling strategy	Random. A priori power calculations aimed to ensure sufficient power to detect more general medium effects of acute stress on the willingness to exert effort within and between experiments, with reduced power to detect more complex higher-order interactions with a similar effect size (e.g., with total n=160: power=0.88 to detect an Experiment×Condition interaction on choice behaviour with effect size $\eta 2$=0.065 at alpha=0.05; power=0.59 to detect a Condition×Effort×Threat interaction on choice behaviour). Specifically, the power analysis was based on ensuring power=0.80 to detect a ~10% difference in overall acceptance of effort between acute stress and control participants at alpha=0.05 (two-tailed) and assuming an SD of 12 in each group (group difference and SD based on previous empirical data investigating the effect of acute stress on an effort-based learning task; https://doi.org/10.1016/j.psyneuen.2021.105646).
Data collection	Data collection<br>- Informed consent was collected using pencil and paper<br>- The timing of saliva samples and vital signs measurements was collected using pencil and paper<br>- Saliva samples were collected using Sarstedt Salivettes (blue cap)<br>- Vital signs were measured using an OMRON M4-I blood pressure meter<br>- Demographics and other questionnaire ratings were collected digitally in Qualtrics (using a computer)<br>- MGF and shock tolerance calibration were collected in Psychopy<br>- MAST procedures were conducted using two computers (to present arithmetic prompts/hand immersion prompts on one screen, and fake video recordings on the other screen)<br>- Task data were collected in Psychopy. For all calibration and task procedures, shocks were administered via a BIOPAC STM100C + STMISO module (administered via gelled electrodes), force was exerted on a BIOPAC TSD121B-MRI dynamometer (connected to a DA100C signal amplifier), and force data were collected/stored via the BIOPAC MP160 module.<br><br>Data were collected with only experimenters present. Experimenters were not blind to study condition due to the condition specific procedures.
Timing	Data collection ran from March/2021 - May/2023

Data exclusions	2 participants were excluded because they failed to pass inclusion/exclusion criteria; 2 participant datasets were incomplete due to hardware and/or saving issues; 1 participant was excluded and discontinued after becoming unwell near the end of the session<br>All criteria were pre-established.<br><br>13 participants across both experiments were classified as inflexible responder. Inflexible responder status definition was pre-defined. Results with and without exclusion of inflexible responders are reported.
Non-participation	4 participants across both experiments stopped participation after being informed about or starting stress-induction procedures
Randomization	Participants were randomly allocated to a condition. Participants that indicated that they had participated in the acute stress condition (in a previous study) were allocated to the control condition.

Reporting for specific materials, systems and methods

We require information from authors about some types of materials, experimental systems and methods used in many studies. Here, indicate whether each material, system or method listed is relevant to your study. If you are not sure if a list item applies to your research, read the appropriate section before selecting a response.

Materials & experimental systems

n/a	Involved in the study
☒	☐ Antibodies
☒	☐ Eukaryotic cell lines
☒	☐ Palaeontology and archaeology
☒	☐ Animals and other organisms
☒	☐ Clinical data
☒	☐ Dual use research of concern

Methods

n/a	Involved in the study
☒	☐ ChIP-seq
☒	☐ Flow cytometry
☒	☐ MRI-based neuroimaging
